# Self‐Efficacy as a Mediator Between Medication Adherence and Self‐Care in Inflammatory Bowel Disease: A Multicenter Cross‐Sectional Study

**DOI:** 10.1111/jocn.70385

**Published:** 2026-05-31

**Authors:** Piergiorgio Martella, Manuele Cesare, Silvia Cilluffo, Alessandro Monaci, Valentina Biagioli, Davide Bartoli, Daniele Napolitano, Antonello Cocchieri

**Affiliations:** ^1^ Neuroscience Department, Neurosurgery Care Management Gemelli IRCCS University Hospital Foundation Rome Italy; ^2^ Department of Biomedicine and Prevention Tor Vergata University Rome Italy; ^3^ A. Gemelli IRCCS University Hospital Foundation Rome Italy; ^4^ Section of Hygiene, University Department of Life Sciences and Public Health Catholic University of the Sacred Heart Rome Italy; ^5^ Department of Biomedical Sciences for Health University of Milan Milan Italy; ^6^ Nursing Direction Paideia International Hospital Rome Italy; ^7^ Department of Medical and Surgical Sciences (DIMEC) University of Bologna Bologna Italy; ^8^ Interdisciplinary Department of Wellbeing, Health and Environmental Sustainability – BeSSA Department Sapienza University of Rome Rieti Italy; ^9^ SITRA and Scientific Direction Rome Italy

**Keywords:** inflammatory bowel disease, medication adherence, self‐care, self‐efficacy

## Abstract

**Aims:**

To examine the role of self‐efficacy in the relationship between medication adherence and self‐care behaviours in patients with Inflammatory Bowel Disease by describing their levels and exploring the interconnections among these variables.

**Design:**

Multicenter, cross‐sectional.

**Methods:**

A total of 452 patients were recruited through consecutive non‐probabilistic sampling across nine Italian outpatient Inflammatory Bowel Disease Units. Data were collected using validated tools: the Morisky Medication Adherence Scale‐8, the Self‐Care Self‐Efficacy Scale, and the Self‐Care of Chronic Illness Inventory. Descriptive statistics, Pearson correlations, and mediation analyses were performed to explore associations and the mediating role of self‐efficacy between medication adherence and self‐care behaviours.

**Results:**

Participants had a mean age of 43.49 years; 50.9% were male, 49.2% had Crohn's disease, and 50.8% had ulcerative colitis. Only 10.2% reported high medication adherence, while most showed medium or low adherence. The mean self‐efficacy score was 74.82. Medication adherence was positively associated with self‐care maintenance, and self‐efficacy statistically accounted for part of this association. Lower levels were observed in self‐care monitoring and management behaviours.

**Conclusions:**

Medication adherence was positively associated with self‐care maintenance, and self‐efficacy partially explained this relationship.

**Implications for Clinical Practice:**

Routine assessment of medication adherence and self‐efficacy may help identify patients at risk of poor self‐care. Interventions aimed at strengthening self‐efficacy, such as motivational interviewing, nurse‐led counselling, and digital monitoring tools, may improve adherence and self‐care maintenance.

**Impact:**

The study addressed low medication adherence and suboptimal self‐care in patients with IBD. Findings support integrating self‐efficacy‐enhancing strategies into multidisciplinary care to improve adherence and self‐care behaviours.

**Patient or Public Contribution:**

Patients completed validated self‐report questionnaires; however, they were not involved in the study design, conduct, analysis, or manuscript preparation.

## Key Points

- Most IBD patients report medium‐to‐low medication adherence, with forgetfulness as the main barrier.
- Medication adherence and Self‐care maintenance share behavioral proximity in IBD.
- Self‐efficacy partially mediated the link between medication adherence and Self‐care maintenance.
- Strengthening self‐efficacy may improve medication adherence and Self‐care in IBD patients.

## 1 Introduction

Inflammatory bowel disease (IBD) is a chronic, progressive, and immune‐mediated disorder affecting the gastrointestinal tract (Clough et al. 2024). Conventional therapeutic approaches have mainly focused on symptom resolution and have proven effective in inducing and maintaining remission while preventing complications in a substantial proportion of patients (Gonczi et al. 2019; West et al. 2023). For this reason, adherence to treatment, defined as “the degree to which a patient's behavior corresponds to the prescriptions agreed with healthcare professionals” (Cleves‐Valencia et al. 2024), is a cornerstone of achieving favourable therapeutic results and minimizing risks related to the disease (Aluzaite et al. 2021; Gohil et al. 2022).

Managing IBD requires a combination of approaches, often involving a mixture of medication regimens (e.g., oral and rectal formulations, subcutaneous injections, and intravenous infusions). These strategies are tailored to the patient's symptoms, disease severity, and treatment response (Aluzaite et al. 2021; Muzammil et al. 2023). However, despite the availability of numerous therapies for IBD and their proven effectiveness, achieving patient medication adherence remains a persistent challenge for public health (Aluzaite et al. 2021; Lasa et al. 2020).

Patients can have difficulties following prescribed regimens due to several factors, including socioeconomic, personal, treatment, health condition, and healthcare‐related barriers (Aluzaite et al. 2021; Cai et al. 2021; Folkvord et al. 2024). A lack of medication adherence in IBD patients not only increases clinical risks, such as recurrence and increased risk of colorectal cancer, but also elevates healthcare costs by increasing the risk of hospitalisation and surgery, as observed in other chronic conditions (Baryakova et al. 2023).

Beyond pharmacological treatment alone, effective IBD management also requires patients to actively engage in daily self‐care behaviours and develop confidence in managing their condition. In this context, self‐care and self‐efficacy have emerged as relevant constructs in chronic disease management and may influence how patients adhere to treatment recommendations and manage their illness over time (Napolitano et al. 2024; Sheehan et al. 2023).

## 2 Background

IBD, which includes Crohn's disease (CD) and Ulcerative Colitis (UC), is characterised by alternating phases of remission and relapse, often associated with symptoms such as abdominal pain, diarrhoea, fatigue, rectal bleeding, and impaired quality of life (Cai et al. 2021; Clough et al. 2024; Le Berre et al. 2020; Spagnuolo et al. 2021). Given the chronic and fluctuating nature of the disease, patients are required not only to adhere to prescribed therapies but also to engage in complex self‐care activities over time.

Self‐care is defined within the Middle‐Range Theory of Self‐Care of Chronic Illness as a process through which individuals maintain health, monitor symptoms, and manage illness‐related changes (Riegel et al. 2019, 2012). This theoretical framework conceptualises self‐care as comprising three dimensions: self‐care maintenance, self‐care monitoring, and self‐care management. Self‐care maintenance includes routine behaviours aimed at preserving health and stability, such as taking medications as prescribed, attending follow‐up visits, or adopting healthy lifestyles. Self‐care monitoring refers to the observation and recognition of symptoms or bodily changes, whereas self‐care management involves the responses implemented when symptoms occur or worsen.

Within this framework, medication adherence can be considered one component of self‐care maintenance, but it does not fully overlap with the broader construct of self‐care. Indeed, self‐care maintenance also includes additional health‐promoting behaviours beyond medication‐taking, while self‐care monitoring and self‐care management involve more complex cognitive and behavioural processes related to symptom interpretation and response. Therefore, although conceptually related, medication adherence and self‐care represent partially distinct constructs.

In chronic illnesses such as heart failure and diabetes, higher levels of self‐care have been associated with improved adherence and better clinical outcomes (Amerzadeh et al. 2024; Krzeminska et al. 2021; Maria et al. 2023), in IBD this relationship remains less thoroughly investigated (Tran and Mulligan 2019).

Self‐efficacy, defined as the confidence in one's ability to perform behaviours necessary to achieve desired outcomes (Sheehan et al. 2023), is considered a central determinant of chronic disease self‐management. Higher self‐efficacy may facilitate the implementation of routine health behaviours, including medication adherence and self‐care practices, by increasing patients' confidence in managing symptoms, treatments, and disease‐related challenges. In IBD, higher self‐efficacy has been associated with lower disease burden and better adjustment to illness (Manzari et al. 2024). In other chronic illnesses, improved self‐efficacy correlates with better medication adherence and adoption of positive health behaviours (Chan 2021; Kara 2022). However, the relationship between self‐efficacy and medication adherence in IBD remains unexplored. While research has shown a positive correlation between self‐efficacy and medication adherence in certain patient populations, such as those with hypertension (Shen et al. 2020), evidence specific to IBD patients is less consistent. For instance, Chen et al. (2023) found no significant association between self‐efficacy and medication adherence among IBD patients, raising questions about the generalisability of this relationship within this population.

Moreover, given the established importance of self‐care and self‐efficacy in chronic disease management, further research is warranted to explore the interrelationships among medication adherence, self‐efficacy, and the self‐care dimensions of maintenance, monitoring, and management in IBD patients. Although self‐efficacy is commonly and historically conceptualised as an antecedent of health behaviours, including medication adherence (Achury‐Saldana et al. 2025; Farley 2020; Khalili Azar et al. 2024), the relationships among these variables may also be reciprocal and context‐dependent (Lee et al. 2024; Napolitano, Martella, and Scaldaferri 2025; Pierobon et al. 2023; Riegel et al. 2019).

## 3 The Study

The aims of this study were threefold: to describe the levels of medication adherence, self‐efficacy, and self‐care among IBD patients; to analyse the relationships between medication adherence, self‐efficacy, and the three dimensions of self‐care, with particular attention to their behavioural interconnections; and to explore the mediating role of self‐efficacy in the relationship between medication adherence and those self‐care dimensions identified as significantly associated with adherence in the correlation analysis.

## 4 Methods

### 4.1 Design

This study represents a secondary analysis of data collected as part of the baseline phase of a multicenter longitudinal observational study, the protocol of which has been previously published (Napolitano et al. 2024), and conducted in accordance with the Strengthening the Reporting of Observational Studies in Epidemiology (STROBE) statement (von Elm et al. 2008). A prior publication from the same dataset examined self‐care behaviours and their sociodemographic and clinical determinants in this sample (Napolitano, Lo Cascio, et al. 2025); the present manuscript addresses a different research question by investigating the interrelationships among medication adherence, self‐efficacy, and self‐care through a mediation analysis. The specific analytical aims of this manuscript were not pre‐specified in the published protocol and were developed following data collection.

### 4.2 Study Setting and Sampling

This study was conducted across nine Italian hospitals in different regions: four university hospitals in Northern Italy, two in Central Italy, and three in Southern Italy. All participating centers were tertiary‐level outpatient IBD clinics operating within the Italian National Health System, which provides a common regulatory and reimbursement framework for IBD care. Nevertheless, differences in local treatment protocols, patient education programmes, nursing support models, and care quality initiatives across sites cannot be ruled out and may contribute to heterogeneity in the findings. Data were collected over a three‐month period, from April to June 2024.

### 4.3 Participants

Participants were recruited through a consecutive non‐probabilistic sampling approach. All IBD patients attending outpatient medical visits during recruitment were invited to participate. Each participant received a set of questionnaires in an envelope. For medication adherence evaluation, participants were asked to complete MMAS‐8 using only their current IBD medications. Written informed consent was obtained for voluntary participation in the study.

### 4.4 Inclusion and Exclusion Criteria

To be considered eligible for the study, patients had to meet the following criteria: be at least 18 years old; have a confirmed diagnosis of IBD; be receiving care at one of the selected outpatient clinics; and provide informed consent to participate. Participants were excluded if they: received an IBD diagnosis within the previous 12 months; suffered from another severe symptomatic chronic disease; and were unable to understand written and spoken Italian.

**FIGURE 1:**
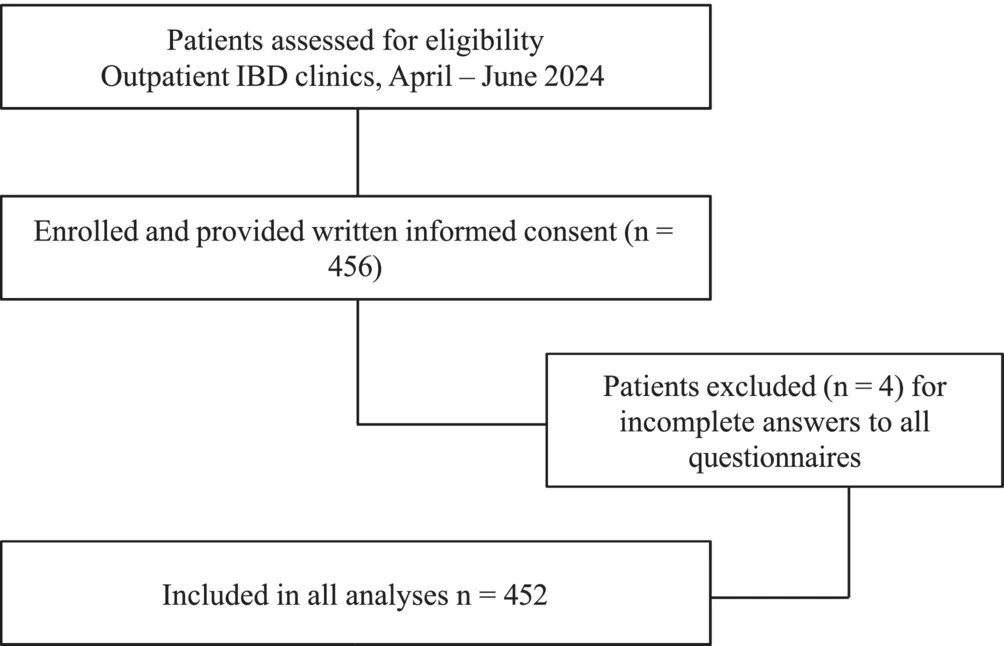
Flowchart illustrating the sampling and exclusion process.

The flow of participants through the study is presented in Figure 1.

### 4.5 Instrument With Validity and Reliability/Data Source

During outpatient visits, patients who agreed to participate received an envelope containing the following adapted questionnaires.

The Sociodemographic questionnaire is a 9‐item instrument to identify participants' sociodemographic characteristics. The items included gender, age, type of pathology, education level, occupation, residence, comorbidities, and years since diagnosis.

The Morisky Medication Adherence Scale‐8 items (MMAS‐8) is a widely used, validated self‐report instrument designed to assess patient adherence to prescribed pharmacological therapies (Berlowitz et al. 2017; Bress et al. 2017). The scale consists of eight items: the first seven are dichotomous (yes/no) questions addressing common barriers to adherence, such as forgetfulness, carelessness, and stopping medication when feeling better or worse, while the eighth item uses a 5‐point Likert scale to evaluate the frequency of medication‐taking behaviour. Each response is scored according to a standardised algorithm, yielding a total score ranging from 0 to 8. Based on established cut‐off points, adherence is classified into three levels: high adherence (score = 8), medium adherence (score 6–7), and low adherence (score < 6). The MMAS‐8 has demonstrated good reliability and predictive validity across various chronic conditions, including IBD, and is particularly useful in both clinical and research settings for identifying patients at risk of non‐adherence. The MMAS‐8 was retained because it captures general adherence‐related behaviours and barriers beyond daily dosing frequency, including forgetfulness, intentional discontinuation, and difficulties in maintaining treatment routines.

The Self‐Care Self‐Efficacy Scale (SC‐SES) is a self‐report instrument designed to assess individuals' confidence in their ability to engage in self‐care behaviours essential to managing chronic illness (Yu et al. 2021). The scale consists of 10 items, each rated on a 5‐point Likert scale from 1 (“not confident at all”) to 5 (“extremely confident”), capturing perceived self‐efficacy in areas such as medication adherence, symptom recognition, lifestyle adjustment, and health‐related decision‐making. Total scores are obtained by summing the responses across items, with higher scores indicating greater self‐efficacy. In the present study, the SC‐SES demonstrated excellent internal consistency, with a Cronbach's alpha of 0.91. This aligns with findings from Yu et al. (2021), where internal consistency ranged from *α* = 0.89 to 0.93 across samples from the United States, Italy, Brazil, and Hong Kong (Yu et al. 2021).

The Self‐Care of Chronic Illness Inventory (SC‐CII) is a theory‐based, self‐report instrument developed to assess self‐care behaviours in individuals with chronic conditions (Napolitano, Biagioli, et al. 2025; Riegel et al. 2012). Grounded in the Middle‐Range Theory of Self‐Care of Chronic Illness (Riegel et al. 2012), the SC‐CII evaluates self‐care across three distinct but interrelated domains: self‐care maintenance (7 items), which includes behaviours aimed at preserving health and physiological stability (e.g., “Take prescribed medicines without missing a dose”); self‐care monitoring (5 items), reflecting the individual's attention to changes in symptoms or body signals (e.g., “Monitor for symptoms”); and self‐care management (6 items), which assesses responses to symptom exacerbation or deterioration (e.g., “Call your healthcare provider for guidance”) (De Maria et al. 2021; Riegel et al. 2018).

Each item is scored on a 5‐point Likert scale ranging from 1 (“never”) to 5 (“always”), with raw scores converted to standardised scores ranging from 0 to 100. Higher scores indicate better self‐care performance, and a cut‐off of ≥ 70 on each subscale has been proposed to define adequate self‐care.

The validated Italian version of the SC‐CII previously tested in IBD patients was used in the present study (Napolitano, Biagioli, et al. 2025). In that validation study, the SC‐CII demonstrated good psychometric properties, with Cronbach's alpha coefficients of 0.81 for self‐care maintenance, 0.86 for self‐care monitoring, and 0.92 for self‐care management. In the current sample, Cronbach's alpha coefficients were 0.84 for maintenance, 0.87 for monitoring, and 0.90 for management, indicating good internal consistency.

### 4.6 Data Analysis

Statistical analyses were conducted to describe the study variables and explore their relationships. Descriptive statistics summarised continuous variables as means and standard deviations (SD) and categorical variables as frequencies and percentages. The normality of the variables was assessed by analysing skewness and kurtosis. Variables with skewness values ±2 or kurtosis values exceeding ±7 were deemed non‐normally distributed (Kim 2013). Pearson correlation coefficients were calculated to explore relationships between medication adherence, self‐efficacy, and the three self‐care dimensions (maintenance, monitoring, and management). A mediation analysis was performed using the PROCESS macro for SPSS (model 4) (Hayes 2013) to examine whether self‐efficacy statistically accounted for the association between medication adherence and the dimensions of self‐care identified as related to medication adherence through correlations (Jung 2021; Rijnhart et al. 2021). The analysis included total, direct, and indirect effects, with bootstrap confidence intervals (95% CI) calculated from 5000 resamples to determine the significance of the mediation pathways (Tibbe and Montoya 2022). The explained variance was assessed using *R* ^2^, and model fit was evaluated using *F*‐statistics. All statistical analyses were conducted using a two‐tailed approach, with significance set at a *p*‐value less than 0.05. Data were analysed using IBM SPSS Statistics version 29 for MacOS (IBM Corp., Armonk, NY, USA).

As this study represents a secondary analysis, no a priori sample size calculation was performed for the present aims. A post hoc power analysis indicated that, for the primary correlation of interest (*r* = 0.120; *N* = 452; *α* = 0.05, two‐tailed), the achieved statistical power was approximately 0.80. The use of 5000 bootstrap resamples in the mediation analysis is consistent with current recommendations for adequate power in indirect effect estimation.

Missing data were assessed prior to analysis. Four participants were excluded due to incomplete questionnaire responses, yielding a final analytical sample of 452 participants. No imputation was performed.

### 4.7 Ethical Considerations

The study was conducted in accordance with the requirements of Good Clinical Practice, the Revised Declaration of Helsinki (World Medical Association 2025), and the General Data Protection Regulation (GDPR) 2016/679 on privacy. Before the enrollment, a written informed consent was requested. Full oral and written information about the study and its purpose was given to patients enrolled. The study protocol was reviewed and approved by the Territorial Ethics Committee (Approval ID: 0023486/23) on August 2, 2023.

## 5 Results

### 5.1 Sociodemographic and Clinical Characteristics of the Study Sample

A total of 452 patients met the inclusion and exclusion criteria and were subsequently enrolled in this study, and their sociodemographic and clinical characteristics were analysed. The mean age was 43.49 years (SD = 16.44), ranging from 18 to 86. The sample was nearly evenly split by gender, with 50.9% identifying as male. Regarding occupation, the majority of patients were paid workers (63.3%). Diagnoses were evenly distributed between CD (49.2%) and UC (50.8%). The mean time since diagnosis was 11.67 years (SD = 9.42), ranging from 1 to 50 years (Table 1).

**TABLE 1:** Sociodemographic and clinical characteristics of the study sample (*N* = 452).

Variable	Statistics	
Age (years) (mean; SD) (range)	43.49 (16.44)	18–86
Gender (*N*; %)		
Male	230 (50.9)	
Female	222 (49.1)	
Occupation (*N*; %)		
Retired	52 (11.5)	
Student	56 (12.4)	
Paid worker	286 (63.3)	
Domestic worker	28 (6.2)	
Unemployed	30 (6.6)	
Type of diagnosis (*N*; %)		
CD	222 (49.2)	
UC	230 (50.8)	
Time since diagnosis (years) (mean; SD) (range)	11.67 (9.42)	1–50

Abbreviations: CD, Crohn's disease; SD, standard deviation; UC, ulcerative colitis.

### 5.2 Medication Adherence, Self‐Efficacy, and Self‐Care Behaviours of the Sample

Patients were screened for medication adherence, self‐efficacy, and self‐care behaviours. The mean medication adherence score in the sample was 5.87 (SD = 1.59). Among participants, the majority (48.4%) demonstrated medium adherence. Regarding current therapy, 71.9% of patients were receiving biological therapy, and 28.1% were receiving other therapies (including 5‐aminosalicylates, immunosuppressants, and corticosteroids). At the item level, the most frequently reported adherence barrier was forgetting to take medication. The mean self‐efficacy was 74.82 (SD = 16.29). Regarding self‐care behaviours, the mean scores were 72.87 (SD = 12.59) for maintenance, 81.24 (SD = 17.79) for monitoring, and 67.70 (SD = 17.01) for management, indicating variability across the sample in different aspects of self‐care (Table 2).

**TABLE 2:** Medication adherence, self‐efficacy, and self‐care behaviours of the sample (*N* = 452).

Variable	Statistics	
Medication adherence (MMAS‐8) (mean; SD) (range)	5.87 (1.59)	1–8
Medication adherence categories (*N*; %)		
Low	187 (41.4%)	
Medium	219 (48.4%)	
High	46 (10.2%)	
Self‐efficacy (SC‐SES) (mean; SD) (range)	74.82 (16.29)	18–100
Self‐care behaviours categories (SC‐CII) (mean; SD) (range)		
Maintenance	72.87 (12.59)	32–100
Monitoring	81.24 (17.79)	20–100
Management	67.70 (17.01)	20–100

Abbreviations: MMAS‐8, Morisky Medication Adherence Scale‐8 item; SC‐CII, Self‐Care of Chronic Illness Inventory; SC‐SES, Self‐Care Self‐Efficacy Scale; SD, standard deviation.

### 5.3 Relationship Between Medication Adherence, Self‐Efficacy, and Self‐Care

The relationship between medication adherence, self‐care dimensions (maintenance, monitoring, and management), and self‐efficacy was evaluated to explore how adherence behaviours align with patients' self‐care practices and confidence in managing their condition. A significant positive Pearson correlation was found between medication adherence and self‐care maintenance (*r* = 0.120; *p* = 0.011). However, no significant correlations were observed between medication adherence and self‐care monitoring (*r* = 0.039; *p* = 0.411) or self‐care management (*r* = 0.001; *p* = 0.975). Finally, medication adherence significantly correlated with self‐efficacy (*r* = 0.114; *p* = 0.015).

### 5.4 Mediating Role of Self‐Efficacy in the Relationship Between Medication Adherence and Self‐Care Maintenance

Medication adherence was positively associated with self‐care maintenance in the total effect model (*β* = 0.9717, *p* = 0.0093) and the direct effect model (*β* = 0.7177, *p* = 0.0465).

**FIGURE 2:**
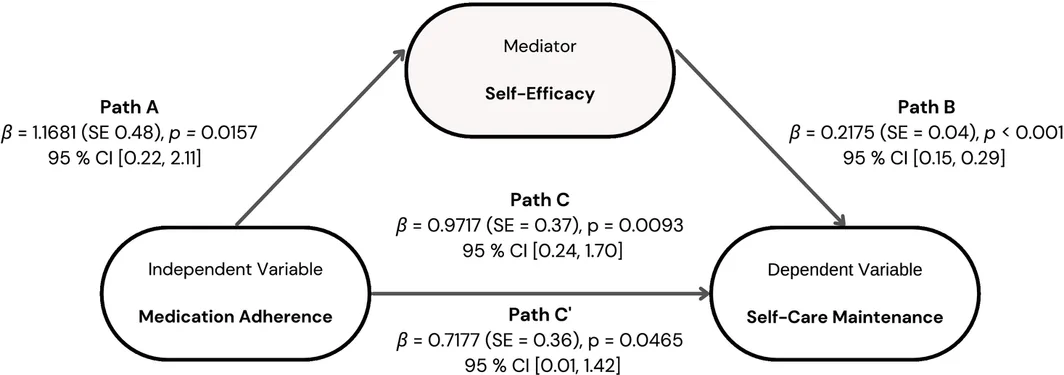
Illustration of mediation analysis model. C = total effect of the independent variable on the dependent variable without considering the mediator; C′ = direct effect of the independent variable on the dependent variable with mediator; the indirect effect is calculated as the difference between the total effect C and the direct effect C′. Model summaries: Path A: *R* = 0.1141; *R* ^2^ = 0.0130; MSE = 262.6214; *F* = 5.8794; df1 = 1; df2 = 446; *p* = 0.0157. Paths B and C′: *R* = 0.3054; *R* ^2^ = 0.0933; MSE = 144.3405; *F* = 22.8927; df1 = 2; df2 = 445; *p* < 0.001. Path C: *R* = 0.1228; *R* ^2^ = 0.0151; MSE = 156.4388; *F* = 6.8303; df1 = 1; df2 = 446; *p* = 0.0093. CI, confidence interval; df, degrees of freedom; MSE, mean squared error; SE, standard error. [Colour figure can be viewed at wileyonlinelibrary.com]

Additionally, a significant indirect effect of medication adherence on self‐care maintenance was observed through self‐efficacy (*β* = 0.2542, 95% CI [0.0497, 0.4954]), indicating that self‐efficacy statistically accounted for part of this association. The standardised indirect effect was small but significant (*β* = 0.0321, 95% CI [0.0063, 0.0622]). The mediation model explained 9.31% of the variance in self‐care maintenance (*R* = 0.3051, *R* ^2^ = 0.0933, *F* = 22.8927, *p* < 0.001). Self‐efficacy was significantly associated with self‐care maintenance after accounting for medication adherence (*β* = 0.2175, *p* < 0.001), while medication adherence also remained significantly associated with self‐care maintenance (*β* = 0.7177, *p* = 0.0465) when both variables were included in the model (Figure 2).

## 6 Discussion

This study pursued three objectives: to describe the levels of medication adherence, self‐efficacy, and self‐care among IBD patients; to analyse the relationships between medication adherence, self‐efficacy, and the three dimensions of self‐care; and to explore the mediating role of self‐efficacy between medication adherence and the self‐care dimensions that were significantly associated with adherence.

The first aim of this study was to assess levels of medication adherence, self‐efficacy, and self‐care in IBD patients. Findings revealed that most participants reported medium or low adherence levels, with only 10.2% achieving high adherence based on MMAS‐8 scores. This aligns with the literature, which reports non‐adherence rates of 33% to 45% among IBD patients (Ghadir et al. 2016; Gohil et al. 2022). Several factors may contribute to this suboptimal adherence, including the chronic nature of IBD, the complexity of therapeutic regimens, and the different drug administration routes (oral, rectal, subcutaneous, intravenous), which can pose logistical and psychological burdens over time (Aluzaite et al. 2021; Muzammil et al. 2023).

Non‐adherence has serious implications: increased risk of relapse, complications, higher healthcare utilisation, and greater corticosteroid use, with associated adverse effects such as infections, osteoporosis, and metabolic disorders (Baryakova et al. 2023; Rhudy et al. 2024). Furthermore, non‐adherence may complicate clinical decisions, as clinicians may misinterpret treatment inefficacy rather than poor adherence or non‐adherence (Papamichael et al. 2022). Therefore, early identification of adherence barriers should be a priority. Integrating motivational interviewing, IBD nurse counselling, and shared decision‐making into routine care may improve adherence and align treatment plans with patient needs and preferences (Mercuri et al. 2024; Napolitano et al. 2020, 2022).

Self‐efficacy levels in our cohort were moderate, suggesting a reasonable but improvable confidence in managing disease‐related tasks. This is relevant, as self‐efficacy plays a critical role in chronic disease self‐management (Chan 2021; Sheehan et al. 2023). While its importance has been well documented in conditions such as diabetes and heart failure, few studies have explored its role in IBD specifically (Munoz Gonzalez et al. 2023; Sheehan et al. 2023). Our data support an association between self‐efficacy and medication adherence and reinforce the relevance of self‐efficacy within broader self‐care processes.

Regarding self‐care, we evaluated the three dimensions proposed by the Middle‐Range Theory of Self‐Care of Chronic Illness (maintenance, monitoring and management) theorised by Riegel et al. (2012). Interestingly, our findings diverge from those reported in other chronic conditions, such as diabetes, where self‐care maintenance generally records the highest score (Fabrizi et al. 2020; Krzeminska et al. 2021). In contrast, IBD patients in our study reported higher scores on self‐care monitoring, possibly reflecting heightened vigilance toward symptom fluctuations, which are a significant concern in this population (Vitello et al. 2024; Volpato et al. 2021). The relatively low scores in the management dimension may indicate a perceived difficulty in acting independently in response to symptoms, particularly in immune‐mediated diseases where professional input is often necessary to guide appropriate responses (Vangeli et al. 2015). The self‐care management score falling below the acceptable threshold highlights a critical area for clinical intervention. Meanwhile, the self‐care maintenance dimension, closely tied to medication adherence, showed moderately favourable scores, indicating that many patients understand the importance of routine behaviours for maintaining disease stability (Chuang et al. 2019; Grandieri et al. 2023; Riegel et al. 2016).

The second aim of this study was to explore associations among variables. We found a statistically significant positive correlation between medication adherence and self‐care maintenance. Patients with higher adherence were more likely to engage in self‐care maintenance behaviours, which include not only following prescribed therapies but also adopting health‐promoting activities such as healthy eating or routine check‐ups (Chuang et al. 2019; Cross et al. 2020; Napolitano, Biagioli, et al. 2025). This association likely reflects the behavioural proximity between medication adherence and routine self‐care activities, as both involve the consistent implementation of prescribed or recommended health‐related actions.

However, no significant correlations were found between adherence and the dimensions of self‐care monitoring and self‐care management. This suggests that while patients may follow treatment plans, they might lack the skills or confidence to identify early warning signs or take appropriate action, underscoring a gap in the more complex, situational components of self‐care (Cadel et al. 2021). This observation supports prior research indicating that self‐care maintenance behaviours are typically the most developed self‐care component in chronic illness populations (Fabrizi et al. 2020; Krzeminska et al. 2021), whereas self‐care monitoring and self‐care management demand greater cognitive, emotional, and behavioural skills (Riegel et al. 2012; Tran and Mulligan 2019). To address this, structured interventions may be needed, ranging from educational programmes and cognitive‐behavioural therapy to digital tools and telemedicine, which can support symptom tracking and early detection (Iizawa et al. 2023; Napolitano, Bozzetti, et al. 2025; Napolitano, Lo Cascio, et al. 2025; Ostlund et al. 2021).

We also observed a significant association between medication adherence and self‐efficacy. This aligns with findings in other chronic conditions (Kara 2022; Maria et al. 2023) and suggests that patients who believe in their ability to manage IBD are more likely to adhere to treatment plans. Targeting self‐efficacy through tailored interventions may therefore improve both medication adherence and self‐care maintenance in this population.

The relationship between self‐efficacy and self‐care dimensions was further examined. A significant correlation emerged only with self‐care maintenance, whereas no association was found with self‐care monitoring and self‐care management. This may reflect the more routine and structured nature of maintenance behaviours, which require less initiative and interpretation than the other two domains (Chuang et al. 2019). Self‐care monitoring and self‐care management often involve interpreting symptoms and making timely and autonomous decisions, abilities that may be underdeveloped in many IBD patients (Lee et al. 2024; Riegel et al. 2021).

Lastly, our third objective focused on the mediating role of self‐efficacy in the relationship between adherence and self‐care. Given the observed correlations, the mediation analysis focused on self‐care maintenance. Results demonstrated that self‐efficacy statistically accounted for part of this association. This pattern is consistent with the role of self‐efficacy in supporting the enactment of routine health behaviours within chronic illness management. In other words, higher levels of self‐efficacy were associated with both medication adherence and self‐care maintenance; however, due to the cross‐sectional design, the temporal or causal direction of these relationships cannot be determined. These findings are consistent with the theoretical framework of self‐care, where self‐efficacy plays a central role in health behaviours, although alternative directional pathways (e.g., self‐efficacy influencing adherence) are equally plausible and supported by previous literature (Riegel et al. 2019).

In conclusion, this study highlights the interrelationship between medication adherence and self‐efficacy in relation to self‐care maintenance in IBD patients. While adherence was associated with routine aspects of disease management, it may not be sufficient to sustain more complex behaviours, such as symptom monitoring and management, which require additional cognitive and psychosocial resources. In this perspective, self‐efficacy may represent a relevant target for interventions aimed at enhancing adherence, promoting patient autonomy, and improving long‐term disease management.

### 6.1 Strengths and Limitations

This study stands out for its multicenter design and large sample size, ensuring good representativeness and statistical power. It is theoretically grounded and provides valuable insights for patient‐centred clinical interventions. However, its cross‐sectional nature prevents causal conclusions. Moreover, medication adherence and self‐care maintenance reflect closely related behavioural processes within chronic illness management, which should be considered when interpreting the observed association. The use of self‐reported questionnaires introduces potential bias, and convenience sampling may lead to selection bias. The lack of a significant association between medication adherence and certain self‐care dimensions suggests a need to refine measurement tools. Furthermore, although all centers operated within the Italian National Health System, potential differences in local treatment protocols, nursing support, and patient education programs across the nine participating hospitals may have introduced contextual variability that could not be fully controlled for. A further limitation concerns the use of the MMAS‐8, which was originally developed to assess adherence to daily oral medications. Given that several IBD treatments are administered on non‐daily schedules (e.g., subcutaneous or intravenous biologics), some items of the scale (e.g., ‘Did you take your medication yesterday?’) may not be fully applicable to all participants. This should be considered when interpreting the medication adherence scores. Finally, the specific analytical aims of this secondary analysis were not pre‐planned in the published protocol.

### 6.2 Recommendations for Further Research

Future research could investigate how caregiver involvement affects the relationship between medication adherence and patient self‐care. Furthermore, future longitudinal studies should examine the temporal and causal direction of the relationships among medication adherence, self‐efficacy, and self‐care, given that the present study's cross‐sectional design does not allow causal inference and that alternative directional pathways remain equally plausible. Finally, additional studies could focus on implementing a digital intervention to assess whether it increases medication adherence and self‐care.

### 6.3 Implications for Clinical Practice

This study underscores the importance of assessing medication adherence and self‐efficacy in IBD patients using simple, validated self‐report tools to identify individuals at risk of non‐adherence or lacking confidence in disease management. The mediating role of self‐efficacy suggests that interventions to strengthen patient confidence, such as motivational interviewing, psychoeducational programs, or nurse‐led counselling with a dedicated IBD nurse, may improve adherence and self‐care maintenance (Knowles and Alex 2020; Rosso et al. 2021; Shinzaki et al. 2022). The low self‐care monitoring and management scores highlight a gap in IBD patients' ability to recognise and act upon symptoms. To address this gap, tailored educational support and the use of digital tools for symptom tracking may be considered. Integrating these strategies within multidisciplinary care models can enhance patient autonomy, reduce disease relapses, and improve long‐term outcomes in IBD management.

## Author Contributions

**Piergiorgio Martella:** conceptualization, methodology, validation, formal analysis, investigation, writing – original draft, writing – review and editing, visualization, project administration, supervision. **Manuele Cesare:** validation, formal analysis, writing – original draft, writing – review and editing, visualization, project administration. **Daniele Napolitano:** conceptualization, validation, resources, visualization, writing – review and editing, project administration. **Alessandro Monaci, Valentina Biagioli, Davide Bartoli, Silvia Cilluffo:** writing – review and editing. **Antonello Cocchieri:** conceptualization, methodology, validation, formal analysis, investigation, writing – original draft, writing – review and editing, visualization, project administration.

## Funding

The authors have nothing to report.

## Disclosure

Statistical statement: (a) The authors have checked to make sure that our submission conforms as applicable to the Journal's statistical guidelines described here. (b) The authors confirm that our submission conforms to the Journal's statistical guidelines. The author team includes statisticians, specifically Manuele Cesare (M.C.), who contributed their statistical expertise to this manuscript. (c) The author(s) affirm that the methods used in the data analyses are suitably applied to their data within their study design and context, and the statistical findings have been implemented and interpreted correctly. (d) The author(s) agree to take responsibility for ensuring that the choice of statistical approach is appropriate and is conducted and interpreted correctly as a condition to submit to the Journal.

## Conflicts of Interest

The authors declare no conflicts of interest.

## Supporting information


**Data S1:** STROBE Statement—Checklist of items that should be included in reports of cross‐sectional studies.

## Data Availability

The data that support the findings of this study are available from the corresponding author upon reasonable request.
